# Optimizing predictive accuracy of the two-component repair model for pediatric TBI: Pediatric-specific parameters, toxicity endpoint harmonization, and dose-rate safeguards

**DOI:** 10.1016/j.ctro.2025.101004

**Published:** 2025-06-27

**Authors:** Ruijie Meng

**Affiliations:** Department of Radiation Oncology, The First Affiliated Hospital of Xi’an Jiaotong University, PR China

**Keywords:** Pediatric TBI, Renal toxicity, Radiation tolerance prediction, Dose-rate effects, Precision radiotherapy

## Abstract

In response to the innovative two-component repair model for pediatric TBI renal toxicity prediction, this letter proposes three key refinements to enhance clinical translation: adopting pediatric-specific radiobiological parameters (e.g., DNA-PKcs dynamics, α/β ratios) to address systematic overestimation of radiation tolerance; harmonizing toxicity endpoints to CTCAE v5.0 ≥Grade 3 criteria to strengthen doseresponse associations and enable precise risk stratification; and implementing institution-specific minimum dose-rate thresholds to mitigate unmodeled vascular susceptibility during low-dose-rate TBI. Collectively, these optimizations will improve predictive accuracy and support personalized radiotherapy for high-risk pediatric cohorts.

To the Editor,

We applaud Eric D. Ehler and colleagues for their seminal development of an innovative two-component repair model. This pioneering work provides the first quantitative analysis of dose-rate and fractionation interval effects on renal toxicity in pediatric Total Body Irradiation (TBI), establishing a novel framework for personalized radiotherapy. To enhance clinical translation and optimize therapeutic decision-making for high-risk pediatric cohorts, we propose three constructive refinements.

First, while the model employs murine-derived renal repair kinetics, human pediatric kidneys exhibit fundamentally distinct radiobiological properties: accelerated cellular turnover, enhanced DNA damage repair capacity mediated by DNA-PKcs upregulation, and a significantly higher clinical α/β ratio than rodent systems [1]. Such interspecies extrapolation systematically overestimates radiation tolerance thresholds, potentially compromising renal function in pediatric patients. We advocate recalibrating repair parameters using pediatric-specific evidence, including functional compensation data from Wilms tumor irradiation cohorts or developmental scaling approaches [2].

Second, conflating CTCAE Grade 1 (subclinical laboratory anomalies) with Grade 4 (intervention-requiring organ failure) events obscures critical dose–response relationships [3]. Restricting endpoints to clinically consequential toxicities (≥Grade 3, denoting medically significant functional impairment) markedly strengthens the association between radiation exposure and renal damage. This endpoint heterogeneity impedes precise risk stratification and may precipitate suboptimal clinical management. Standardization using CTCAE v5.0 ≥Grade 3 criteria with stratified risk reporting is essential for evidence-based decision-making.

Third, the model's substantial toxicity underestimation at low dose rates—particularly in single-fraction TBI—stems from unmodeled vascular endothelial susceptibility during protracted irradiation. To address this gap, we advocate implementing institution-specific minimum dose-rate thresholds to mitigate microvascular injury while developing adaptive dose-reduction protocols responsive to real-time dosimetric variability.

These refinements will significantly augment the model's utility in personalizing pediatric TBI. We commend the authors' transformative contribution and anticipate its pivotal role in advancing precision radiotherapy.

## CRediT authorship contribution statement

**Ruijie Meng:** Conceptualization, Methodology, Formal analysis.

## Funding

The authors received no specific funding.

## Declaration of competing interest

The authors declare that they have no known competing financial interests or personal relationships that could have appeared to influence the work reported in this paper.
